# Dissemination of integrons and carbapenemase-encoding genes among multidrug resistant *Proteus mirabilis* isolated from urinary tract infections in Egypt

**DOI:** 10.1186/s12879-025-12447-4

**Published:** 2026-01-27

**Authors:** Shaimaa Zaki, Dalia N. Kotb, Soha S. Abdelrahim

**Affiliations:** Department of Medical Microbiology and Immunology, Faculty of Medicine, Minia University, Minia, 61511 Egypt

**Keywords:** Proteus mirabilis, Carbapenemases, AmpC, Integrons, Urinary tract infection, Egypt

## Abstract

**Background:**

*Proteus mirabilis (P. mirabilis)* is an opportunistic pathogen responsible for various community-acquired and nosocomial infections, particularly urinary tract infections (UTIs). Rising resistance to broad-spectrum antibiotics, including carbapenems, is narrowing treatment options and poses a major public health concern. This study aimed to investigate the antimicrobial susceptibility patterns of *P. mirabilis* isolates obtained from UTI patients. Then, to assess the molecular determinants of carbapenem-resistant isolates with a focus on the role of integrons in resistance gene dissemination.

**Methods:**

A total of 101 *P. mirabilis* isolates were recovered from 600 urine samples collected from both inpatients and outpatients at Minia University Hospitals, Egypt. The disc diffusion method was utilized for phenotypic identification and to determine the antimicrobial susceptibility profiles of carbapenem-resistant isolates. Subsequently, conventional PCR was performed to screen for eleven carbapenemase-encoding genes, AmpC β-lactamase genes, and integrons.

**Results:**

Among the 101 isolates, 57 (56.4%) were resistant to imipenem, with the majority recovered from inpatients (80.7%) and catheterized patients (56.1%). Carbapenem-resistant isolates exhibited significantly higher resistance rates to ceftazidime (87.7% vs. 27.3%), aztreonam (50.9% vs. 18.2%), and gentamicin (15.8% vs. 0%) compared to carbapenem-sensitive isolates (*p* < 0.05). Multidrug resistance (MDR) was identified in 93% of the imipenem-resistant isolates. Molecular analysis revealed a predominance of class B metallo-β-lactamases, with, *bla*_VIM−1_ (78.9%) and *bla*_NDM_ (24.6%) being the most common. Additionally, class A and class D carbapenemase genes were detected, although at comparatively lower frequencies. Out of 101 isolates, 39 (38.6%) were identified as ESBL producers, of which 13 (33%) were positive for either the *bla*_TEM_ or *bla*_SHV_ gene. AmpC β-lactamase gene (*bla*_FOX_) was identified in 7.9% of isolates. Integrons were widespread, with *intI1* present in 91.2% and *intI2* in 47.4% of resistant isolates. Notably, co-carriage of three or more carbapenemase genes was observed in 64.9% of resistant isolates, all of which exhibited MDR phenotypes.

**Conclusion:**

Carbapenem resistance in *P. mirabilis* is highly prevalent and strongly linked to MDR, integron carriage. This reflects the ongoing evolution of antibiotic resistance and the key function of integrons in spreading resistance genes. The detection of isolates carrying several carbapenemase genes simultaneously is particularly worrisome. These findings highlight the importance of implementing antimicrobial stewardship alongside ongoing molecular surveillance to effectively track and control the spread of resistant strains.

**Supplementary Information:**

The online version contains supplementary material available at 10.1186/s12879-025-12447-4.

## Background

*Proteus* is a motile, Gram-negative, facultatively anaerobic bacterium belonging to the family *Enterobacteriaceae* [1]. As commensal inhabitants of the intestinal microbiota in both humans and animals, these bacteria are capable of surviving and persisting in diverse environmental reservoirs, including sewage, water, and soil. The most clinically significant pathogenic species within this genus is *P. mirabilis*. Infections caused by *P. mirabilis* can be both community- and hospital-acquired and are commonly associated with catheter-associated urinary tract infections (CAUTIs), particularly cystitis and pyelonephritis. The organism is also implicated in kidney stone formation and, less frequently, in wounds, burns, ophthalmic, and intestinal infections [2].

The rapid emergence of antimicrobial resistance among Gram-negative bacteria is rendering the treatment of infectious diseases increasingly challenging. *Proteus* species possess intrinsic resistance to several antibiotics, including colistin, tetracycline, and nitrofurantoin. However, *Proteus* species lack chromosomally encoded β-lactamases and thus do not exhibit inherent resistance to β-lactam antibiotics; any β-lactam resistance observed is solely due to acquired genetic determinants [3].

*P. mirabilis* exhibits resistance to expanded-spectrum cephalosporins (ESC) through the production of extended-spectrum β-lactamases (ESBLs) and plasmid-mediated AmpC β-lactamases. Plasmid-encoded AmpC genes, including *MOX*, *FOX*, *DHA*, and *CIT*, have been identified on plasmids disseminating among *Enterobacteriaceae*. Transmissible plasmids have acquired genes encoding AmpC enzymes, enabling the spread of these resistance determinants to bacterial species that naturally lack the chromosomal *bla*_AmpC_ gene, such as *P. mirabilis* [4].

Carbapenems are considered the first-line treatment for infections due to ESC-resistant *P. mirabilis*. Resistance to carbapenems in *P. mirabilis* arises from the production of carbapenemase enzymes classified in Ambler classes A, B, and D. Additionally, resistance may arise from porin loss, overexpression of efflux pumps, and alterations in penicillin-binding proteins [5]. Class A carbapenemases in *P. mirabilis* include plasmid-encoded enzymes such as Klebsiella pneumoniae carbapenemase (KPC) (KPC-2 to KPC-13) and Guiana extended spectrum (GES), which hydrolyze carbapenems and are partially inhibited by clavulanic acid [6]. Class B carbapenemases hydrolyze carbapenems but are inhibited by ethylenediaminetetraacetic acid (EDTA). The most prevalent metallo-β-lactamase families include New Delhi metallo-β-lactamase 1 (NDM-1), Imipenem-resistant Pseudomonas (IMP)-type carbapenemases, Verona integron-encoded metallo-β-lactamase (VIM), German imipenemase (GIM), and Seoul imipenemase (SIM). Class B Carbapenemases genes are frequently located within integrons and gene cassettes, facilitating their dissemination and contributing to the spread of carbapenem resistance among Gram-negative bacteria [5]. Class D carbapenemases are serine β-lactamases that exhibit poor inhibition by EDTA or clavulanic acid. They belong to the OXA-type enzymes, such as OXA-48 and OXA-23. OXA carbapenemases are notable for their ability to mutate rapidly and disseminate among bacterial populations [7].

The rising incidence of carbapenem-resistant *P. mirabilis* poses a significant threat to the effective management of UTIs, particularly in clinical settings. This study aims to characterize the antimicrobial resistance patterns of *P. mirabilis* isolates from patients with UTIs, with a specific focus on elucidating the molecular mechanisms underlying carbapenem resistance. Furthermore, the research will examine the presence of integron genes, which play a critical role in the acquisition and dissemination of antimicrobial resistance. The findings will contribute to a deeper understanding of resistance mechanisms and support the development of effective antimicrobial stewardship strategies and infection control measures.

## Methods

### Study design

This cross-sectional study included 101 *P. mirabilis* isolates obtained from 600 urine specimens collected randomly from 330 inpatients and 270 outpatients at Minia University Hospitals. Inclusion criteria of patients comprised adults aged 18 years and above presenting with clinical features of UTI, including suprapubic pain, bacteriuria, and pyuria. Inpatients were considered eligible if symptoms appeared 48 h after hospital admission, indicating hospital-acquired infection. Exclusion criteria involved patients with a recent history of antibiotic use before sample collection. This study was performed in the Microbiology and Immunology Laboratories of the Faculty of Medicine of Minia University, between July 2024 and March 2025. The study was conducted in accordance with the ethical principles outlined in the Declaration of Helsinki. Informed written consent was obtained from all participants before inclusion in the study. Ethical approval was granted by the Ethical Committee of the Faculty of Medicine, Minia University, with Approval No.1064/03/2024.

### Bacterial isolation

Urine specimens were collected using sterile containers under fully aseptic conditions and transported to the bacteriology laboratory within two hours of collection. Urine samples from catheterized patients were obtained by first cleaning the catheter tubing with alcohol, then aspirating the urine using a sterile needle and syringe through a sterile puncture site. Samples were centrifuged, and the sediments were cultured on chromogenic agar (CHROMagar™ Orientation, Paris, France), then incubated at 37 °C for 24 h. Cultures yielding bacterial growth were initially identified by their typical colony morphology on nutrient agar and MacConkey’s agar media (Oxoid, UK). *P. mirabilis* isolates were identified based on colony morphology, microscopic examination, and standard biochemical tests, including urease, catalase, and motility positivity; glucose fermentation and H₂S production; and negative reactions for indole and oxidase. Confirmed *P. mirabilis* pure colonies were inoculated in Trypticase soy broth (Oxoid, UK), incubated 24 h at 37 °C, then mixed with sterilized glycerol 20% and stored at − 20 °C for further examination [8].

### Antibiotic susceptibility testing

The Kirby-Bauer disc diffusion technique was used to detect antibiotic susceptibility, utilizing Muller-Hinton agar (Oxoid, U.K.) according to the Clinical Laboratory Standard Institute (CLSI) guidelines, 2023 [9]. The following ten antimicrobial disks were used: Imipenem (IPM) 10 µg, Amoxicillin/clavulanic acid (AMC) 20/10µg, Ceftriaxone (CRO) 30 µg, Ceftazidime (CAZ) 30 µg, Cefotaxime (CTX) 30 µg, Ciprofloxacin (CIP) 5 µg, Cefoxitin (FOX) 30 µg, Aztreonam (ATM) 30 µg, Amikacin (AK) 30 µg, and Gentamicin (CN) 10 µg (Thermo Scientific™, UK). Isolates exhibiting resistance to three or more classes of antimicrobial agents were classified as MDR [10]. *Escherichia coli* (ATCC 25922) was used as a quality control strain.

ESBL production was determined using the double-disk synergy test (DDST) with ceftazidime (30 µg) and cefotaxime (30 µg) disks placed adjacent to an amoxicillin–clavulanic acid disk, in accordance with CLSI (2023) guidelines [9]. Isolates showing the characteristic “keyhole” zone of inhibition between the β-lactam and β-lactamase inhibitor disks were interpreted as presumptive ESBL producers.

### DNA extraction

Genomic DNA was extracted from 101 phenotypically confirmed *P. mirabilis* isolates using the modified boiling technique described by Dashti et al. (2009) [11]. For each strain, three to five colonies were picked and inoculated into tryptone soya broth for overnight incubation. Following incubation, 1.5 ml of the culture was centrifuged at 12,000 rpm for 5 min at 4 °C. After discarding the supernatant, the cell pellet was resuspended in 200 µl of sterile distilled water. This mixture was then subjected to heat treatment in a boiling water bath for 10 min to lyse the cells and release the DNA. The lysate was subsequently cooled on ice for 20 min, followed by a second centrifugation using the same speed and temperature. The resulting clear supernatant, containing the extracted genomic DNA, was carefully transferred to a sterile microcentrifuge tube and stored at − 20 °C for future PCR applications.

### Conventional PCR

#### Detection of carbapenem resistance genes

*P. mirabilis* isolates exhibiting resistance to Imipenem were subjected to molecular screening for 11 carbapenemase-encoding genes using conventional PCR with gene-specific primers listed in Table S1 in the supplementary file. The targeted genes included: *bla*_KPC_, *bla*_GES_, *bla*_PER_, *bla*_VEB_, *bla*_IMP1_, *bla*_VIM1_, *bla*_SIM_, *bla*_GIM_, *bla*_NDM_, *bla*_OXA− 48_, and *bla*_OXA− 23_. A total reaction volume of 25 µL was used for the PCR assay of each target gene, consisting of 5 µL of extracted DNA, 12.5 µL of ready-to-use PCR Master Mix (Applied Biosystems™, USA), 1 µL of each specific primer, and 5.5 µL of nuclease-free water. PCR amplification was conducted under the following thermal cycling conditions: an initial denaturation step at 94 °C for 5 min, followed by 30 cycles consisting of denaturation at 94 °C for 1 min, annealing at gene-specific temperatures, and extension at 72 °C for 45 s; concluding with a final extension at 72 °C for 10 min. Details of the primer sequences, annealing temperatures, and expected product sizes for each target gene are provided in Table S1 of the supplementary file.

#### Detection of ESBL genes

Conventional PCR was performed for phenotypically confirmed ESBL-producing isolates to detect two ESBL genes, *bla*_TEM_ and *bla*_SHV_, using their specific primers. The PCR conditions were as follows: initial denaturation at 95 °C for 5 min; 35 cycles of denaturation at 95 °C for 1 min, annealing at 51 °C for *bla*_TEM_ and 55 °C for *bla*_SHV_ for 1 min each, and extension at 72 °C for 1 min; followed by a final extension at 72 °C for 10 min. The primer sequences and expected amplicon sizes for both genes are listed in Table S1 of the supplementary file.

#### Detection of plasmid-mediated AmpC β-lactamase genes

The detection of AmpC β-lactamase activity was performed using the disc diffusion assay, employing a cefoxitin (30 µg) disc. Isolates demonstrating an inhibition zone diameter of 18 mm or smaller were identified as suspected AmpC β-lactamase producers. Multiplex PCR was conducted to screen plasmid-encoded AmpC β-lactamase genes, including MOX, CIT, DHA, and FOX [12]. PCR amplification was performed under the following thermal cycling conditions: an initial denaturation at 95 °C for 3 min, followed by 30 cycles of denaturation at 94 °C for 45 s, annealing at 64 °C for 45 s, and extension at 72 °C for 1 min, with a final extension at 72 °C for 5 min. The primer sequences and expected product sizes for the four target genes are summarized in Table S1 of the supplementary file.

#### Detection of integron genes

All the *P. mirabilis* isolates were screened for the presence of integron classes 1,2, and 3 [13]. The sequences of primers and the annealing temperatures used for the detection of *intI1*, *intI2*, and *intI3* are shown in Table S1 of the supplementary file. PCR products were electrophoresed on a 2% agarose gel and detected using a UV transilluminator (Biometra, Germany).

### Statistical analysis

All collected data were analyzed using IBM SPSS software (version 23.0). A chi-square test was used for the comparison between the groups, and the results were considered significant only if the P value was < 0.05.

## Results

### Identification of *P. mirabilis* strains

A total of 101 *P. mirabilis* isolates were recovered from 600 urine samples, representing 16.8% of the total. The majority of isolates were from inpatients (78/101, 77.2%), while 23/101 (22.8%) were from outpatients. Among the total urine samples, the isolation rate was 23.6% (78/330) for inpatients and 8.5% (23/270) for outpatients. The isolates were collected from various departments of Minia University Hospitals. Identification of *P. mirabilis* was based on colony morphology on chromogenic agar, where isolates appeared as orange-brown colonies with a surrounding brownish halo. Further confirmation was achieved through standard biochemical tests, including positive results for urease, catalase, motility, glucose fermentation, and hydrogen sulfide (H₂S) production, along with negative reactions for indole and oxidase.

### Characterization of carbapenem-resistant *P. mirabilis*

Out of the 101 *P. mirabilis* isolates, 57 (56.4%) were resistant to imipenem, including 10 isolates (17.5%) that exhibited intermediate resistance. Carbapenem-resistant strains were predominantly isolated from inpatients (46/57, 80.7%) and patients undergoing catheterization (32/57, 56.1%). Notably, 71.9% (41/57) of the resistant isolates were recovered from female patients, including 8 isolates obtained from pregnant women. The distribution of carbapenem-resistant isolates across hospital departments was as follows: 17 (29.8%) from the urology department, 16 (28.1%) from gynecology, 12 (21.1%) from the intensive care unit (ICU), 8 (14%) from surgery, and 4 (7%) from neurology. Clinically, 33 patients (57.9%) with carbapenem-resistant isolates reported dysuria, and 39 (68.4%) presented with fever, as detailed in Table 1.

Antibiotic susceptibility analysis revealed statistically significant differences between carbapenem-resistant (CR) and carbapenem-sensitive (CS) *P. mirabilis* isolates for several antimicrobial agents. Resistance to ceftazidime was significantly higher among CR isolates (87.7%) compared to CS isolates (27.3%) (*p* < 0.001). Similarly, resistance to aztreonam (50.9% vs. 18.2%; *p* = 0.001) and gentamicin (15.8% vs. 0%; *p* = 0.006) was significantly associated with carbapenem resistance. Interestingly, resistance to cefotaxime was more prevalent among CR isolates (43.9%) than CS isolates (22.7%), with a statistically significant difference (*p* = 0.027). Overall, gentamicin and amikacin were the most effective agents against carbapenem-resistant isolates, followed by cefoxitin and ciprofloxacin. A significantly higher prevalence of MDR was observed among carbapenem-resistant isolates compared to carbapenem-sensitive ones, with 53 out of 57 (93%) CR isolates classified as MDR (*p* < 0.001) (Table 1).

### Detection of ESBL-producing *P. mirabilis*

All *P. mirabilis* isolates were phenotypically tested for ESBL production using the double-disk synergy test (DDST), and 39 out of 101 isolates (38.6%) were identified as ESBL producers. Among these, 22 isolates were also resistant to carbapenems. Table 1 revealed that 13 out of 39 (33%) were positive for either the *bla*_TEM_ or *bla*_SHV_ gene. The *bla*_TEM_ gene was present in 11 strains, 7 of which were carbapenem-resistant. The *bla*_SHV_ gene was detected in only 2 strains, and both were carbapenem-resistant. Notably, all ESBL gene–positive isolates were obtained from inpatients and carried the *intI1* gene. All of which exhibited MDR phenotypes.

**Table 1:** Patient characteristics, clinical data, percentage of antibiotic resistance patterns, and molecular genes among carbapenem-resistant and carbapenem-sensitive *P. mirabilis* isolates

Variable	Carbapenem susceptibility of *P*. mirabilis isolates					*P* value
		Sensitive		Resistant		
	Total (101)	Count (44)	%	Count (57)	%	
Patient	Outpatient (23)	12	27.3%	11	19.3%	0.3
	Inpatient (78)	32	72.7%	46	80.7%	
Pregnancy	Yes (10)	2	4.5%	8	14.0%	0.1
Department	G&O (31)	15	34.1%	16	28.1%	0.4
	ICU (22)	10	22.7%	12	21.1%	
	Surgery (14)	6	13.6%	8	14.0%	
	Neurology (10)	6	13.6%	4	7.0%	
	Urology (24)	7	15.9%	17	29.8%	
Dysuria	Yes (59)	26	59.1%	33	57.9%	0.9
Fever	Yes (71)	32	72.7%	39	68.4%	0.6
Catheterization	Yes (54)	22	50.0%	32	56.1%	0.5
Gender	Male (39)	23	52.3%	16	28.1%	0.013*
	Female (62)	21	47.7%	41	71.9%	
Cefotaxime	Resistant (35)	10	22.7%	25	43.9%	0.027*
Cefoxitin	Resistant (27)	9	20.5%	18	31.6%	0.21
Amikacin	Resistant (17)	5	11.4%	12	21.1%	0.1
Ceftazidime	Resistant (62)	12	27.3%	50	87.7%	0.0001*
SXT	Resistant (82)	38	86.4%	44	77.2%	0.2
AMC	Resistant (73)	28	63.6%	45	78.9%	0.08
Ciprofloxacin	Resistant (41)	19	43.2%	22	38.6%	0.642
Azetronam	Resistant (37)	8	18.2%	29	50.9%	0.001*
Gentamicin	Resistant (9)	0	0.0%	9	15.8%	0.006*
Ceftriaxone	Resistant (33)	10	22.7%	23	40.4%	0.061
DDST	Positive (39)	17	38.6%	22	38.6%	0.9
MDR	Positive (72)	19	43.2%	53	93.0%	0.0001*
*bla*_*TEM*_	Positive (11)	4	9.1%	7	12.3%	0.6
*bla*_*SHV*_	Positive (2)	0	0.0%	2	3.5%	0.2
AmpC (*bla*_FOX_)	Positive (8)	4	9.1%	4	7.0%	0.7
*intI1*	Positive (93)	40	90.9%	53	93.0%	0.4
*intI2*	Positive (65)	38	86.4%	27	47.4%	0.0001*
*intI3*	Positive (0)	0	0%	0	0%	**-**

AMC (Amoxicillin/clavulanic acid); SXT (Sulfamethoxazole/trimethoprim); DDST (double-disk synergy test); MDR (Multidrug resistant)

* (significant P value)

### Detection of carbapenemase-encoding genes among the carbapenem-resistant P. mirabilis

**Fig. 1:**
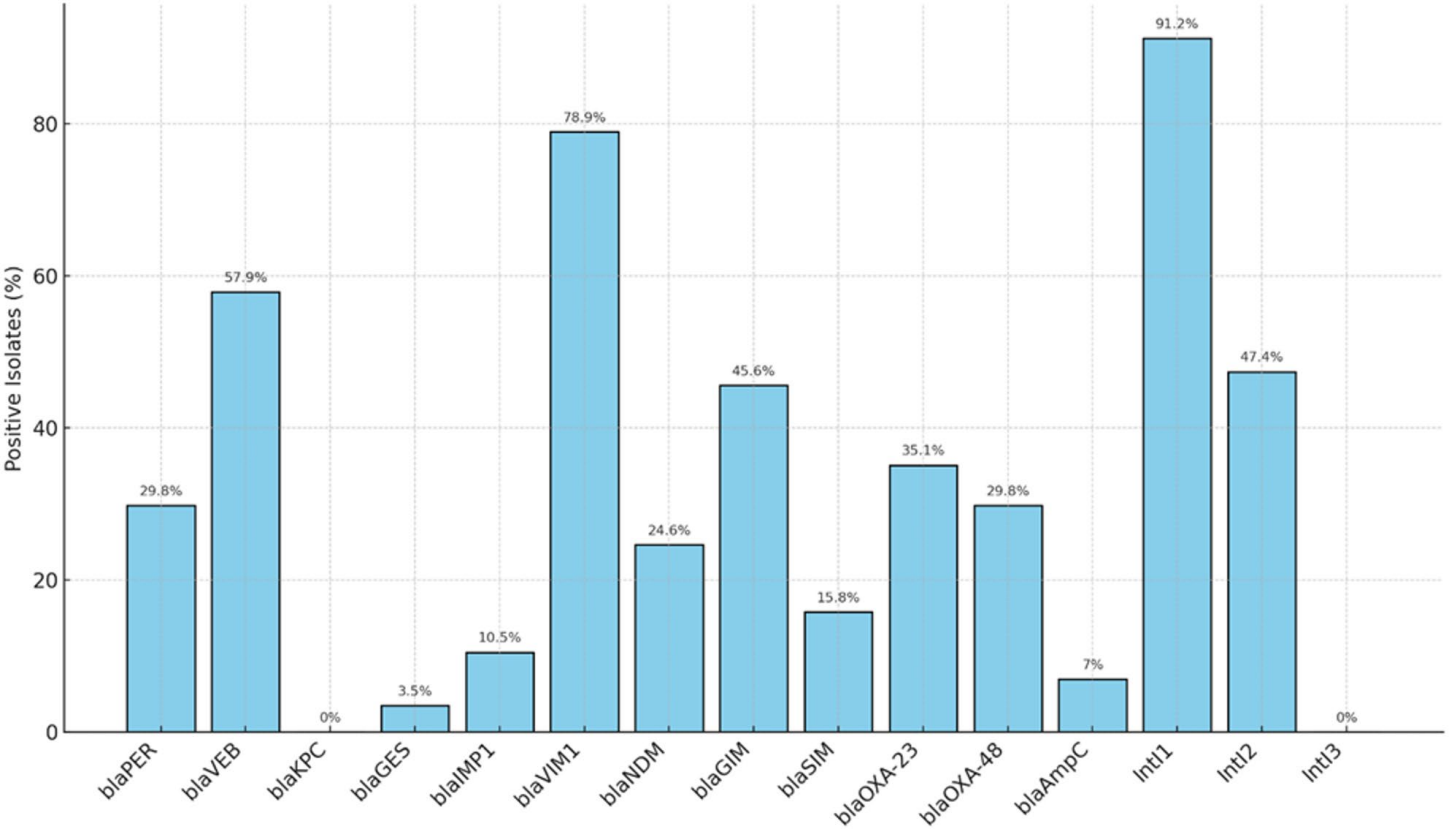
The frequencies of the studied genes among carbapenem-resistant *P. mirabilis* isolates

All 57 carbapenem-resistant *P. mirabilis* isolates were examined for the detection of 11 carbapenem resistance genes by conventional PCR. The frequencies of the studied genes among all isolates are shown in Fig. 1. Class B metallo-β-lactamase (MBL) genes were the most frequently detected, with 51 isolates (89.5%) carrying at least one Class B gene. The most prevalent was *bla*_VIM1_, identified in 78.9% of isolates, followed by *bla*_GIM_ (45.6%), *bla*_NDM_ (24.6%), and *bla*_IMP1_ (10.5%). Class A carbapenemase genes were detected in 43 isolates (75.4%). Among these, *bla*_VEB_ was the most common (57.9%), followed by *bla*_PER_ (29.8%), while *bla*_GES_ was detected in only 3.5% of isolates. The *bla*_KPC_ gene was not detected in any of the isolates. Class D β-lactamase genes (oxacillinases) were identified in 35 isolates (64.8%). Specifically, *bla*_OXA−23_ was found in 35.1% of isolates, and bla_OXA−48_ was found in 29.8% of isolates (Table 2).

**Fig. 2:**
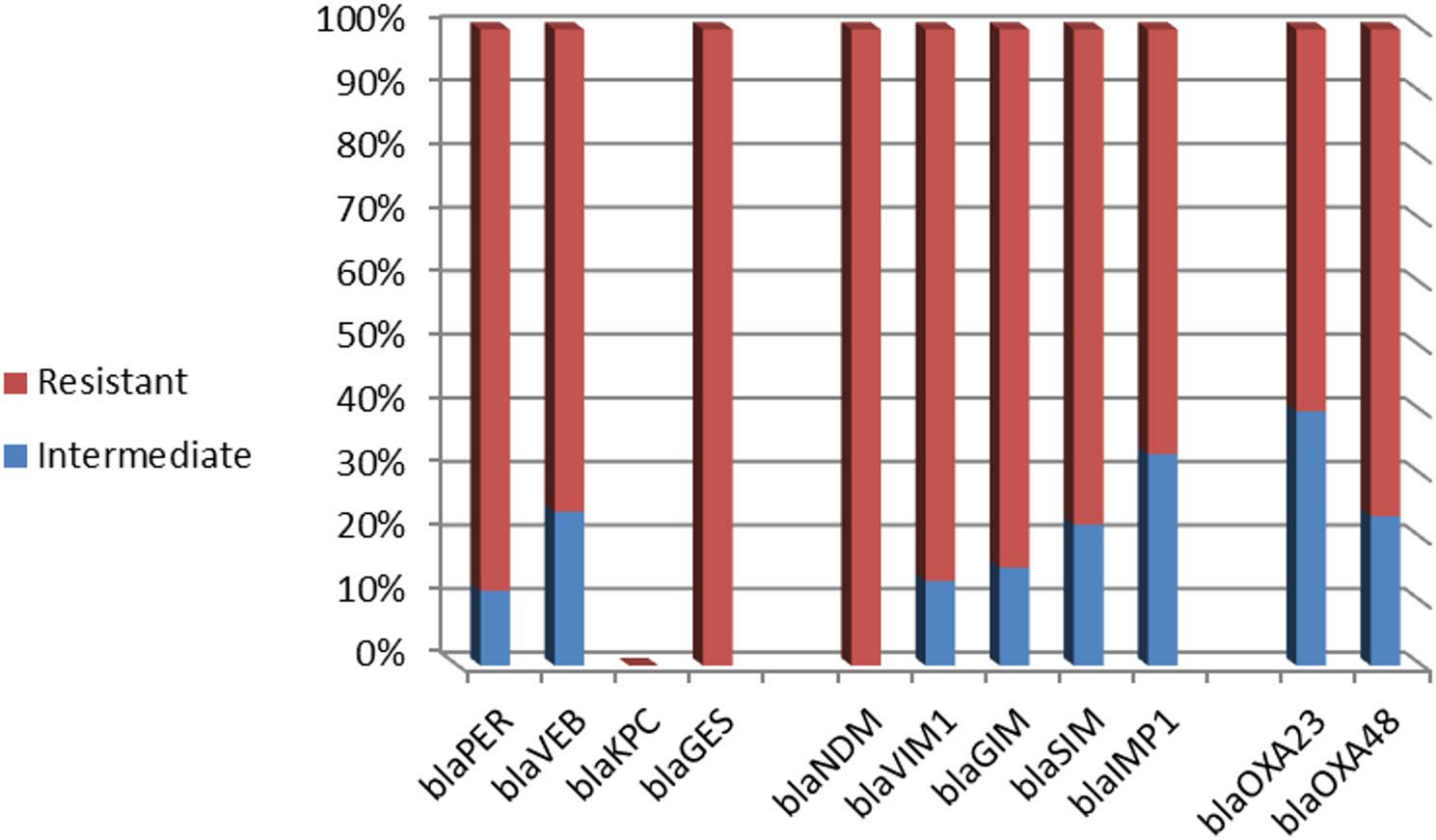
Frequencies of carbapenemase-encoding genes among imipenem-resistant and intermediate-resistant *P. mirabilis* isolates

Based on imipenem susceptibility, the frequencies of carbapenemase-encoding genes among resistant and intermediate-resistant *P. mirabilis* isolates are illustrated in Fig. 2. All these genes were more frequently detected in isolates exhibiting full resistance to imipenem compared to those with intermediate resistance. Notably, *bla*_NDM_ and *bla*_GES_ were not identified in any of the intermediate-resistant isolates. However, *bla*_OXA−23_ was the most detected gene among the intermediate-resistant group.

Notably, *bla*_NDM_ was detected exclusively among inpatient isolates, with a statistically significant association (*p* = 0.035). In contrast, *bla*_OXA−23_ was significantly more prevalent in isolates from outpatients than in those from inpatients (*p* = 0.006). Although Class B and Class D carbapenemase genes were more frequently detected in isolates from catheterized patients than in those from non-catheterized individuals, the difference was not statistically significant (Table 2).

### Detection of AmpC β-lactamase genes

Among the 27 isolates phenotypically identified as AmpC β-lactamase producers based on cefoxitin resistance, only 8 / 27 (29.6%) were confirmed to carry AmpC genes by multiplex PCR. Among all 101 *P. mirabilis* isolates, these eight AmpC-positive isolates accounted for 7.9%. Of the tested AmpC genes, *fox* was the only gene detected, present in all 8 PCR-positive isolates, while *DHA*, *CIT*, and *MOX* genes were not detected in any of the strains. Notably, carbapenemase-encoding genes were co-detected in 4 out of these 8 *fox*-positive isolates (50%).

**Table 2:** Frequency of resistance genes and integrons among carbapenem-resistant *P. mirabilis* isolates in relation to patient characteristics and catheterization status

Gene		Total	Outpatient	Inpatient	*P* value	Cathetrized	Non-Catheterized	*P* value
		*N* = 57	*N* = 11	*N* = 46		*N* = 32	*N* = 25	
Class A β-lactamases	*bla*_PER_	17(29.8%)	3(27.3%)	14(30.4%)	0.8	8(25%)	9(36%)	0.3
	*bla*_VEB_	33 (57.9%)	7(63.6%)	26(56.5%)	0.6	18(56.3%)	15(60%)	0.7
	*bla*_KPC_	0 (0%)	0	0	0	0	0	0
	*bla*_GES_	2(3.5%)	0 (0%)	2(4.3%)	0.4	2(6.3%)	0(0%)	0.2
Class B metallo β-lactamases	*bla*_IMP1_	6 (10.5%)	2(18.2%)	4(8.7%)	0.3	4(12.5%)	2(8%)	0.5
	*bla*_VIM1_	45 (78.9%)	11(100%)	34(73.9%)	0.05	26(81.3%)	19(76%)	0.6
	*bla*_NDM_	14 (24.6%)	0(0%)	14(30.4%)	0.035*	10(31.3%)	4(16%)	0.1
	*bla*_GIM_	26 (45.6%)	6(54.5%)	20(43.5%)	0.5	14(43.8%)	12(48%)	0.7
	*bla*_SIM_	9 (15.8%)	3(27.3%)	6(13%)	0.2	6(18.8%)	3(12%)	0.4
Class D β-lactamases (oxacillinases)	*bla*_OXA−23_	20 (35.1%)	2(18.2%)	18(39.1%)	0.1	14(43.8%)	6(24%)	0.1
	*bla*_OXA−48_	17 (29.8%)	7(63.6%)	10(21.7%)	0.006*	10(31.3%)	7(28%)	0.7
Cephalosporinase (AmpC)	*bla*_FOX_	4 (7%)	0 (0%)	4 (8.7%)	0.3	4 (12.5%)	0 (0%)	0.06
Integrons	*IntI1*	52 (91.2%)	8 (72.7%)	44 (95.7%)	0.016*	30 (93.8%)	22 (88%)	0.4
	*IntI2*	27 (47.4%)	3 (27.3%)	24 (52.2%)	0.013*	20 (62.5%)	7 (28%)	0.01*

*(significant P value)

### Detection of integrons & Coexistence of carbapenemase-encoding genes and integrons among carbapenem-resistant *P. mirabilis*

The presence of *intI1*, *intI2*, and *intI3* genes was used to identify class 1, class 2, and class 3 integrons, respectively, as indicators of gene transfer potential. Class 1 integrons (*intI1*) were highly prevalent among *P. mirabilis* isolates, detected in 94 out of 101 (93.1%) isolates. Class 2 integrons (*intI2*) were also commonly identified, present in 65 isolates (64.4%), while *intI3* was not detected in any of the samples. Among the 57 carbapenem-resistant isolates, all carried at least one integron gene. Specifically, 30 isolates (52.6%) harbored *intI1* alone, 5 isolates (8.8%) carried only *intI2*, and 22 isolates (38.6%) carried both *intI1* and *intI2*. Overall, *intI1* was present in 52 of the 57 (91.2%) carbapenem-resistant isolates, while *intI2* was detected in 27 (47.4%). A statistically significant association was found between the presence of *bla*_GES_ and class 2 integrons, as *bla*_GES_ was detected exclusively in isolates carrying *intI2* (*p* = 0.000). Additionally, strong associations were observed between integron carriage and the *bla*_SIM_ (*p* = 0.001) and *bla*_GIM_ (*p* = 0.002) genes. Notably, *bla*_SIM_ was predominantly found in isolates harboring both *intI1* and *intI2*, whereas *bla*_GIM_ was more frequently associated with *intI1*-positive isolates (Table 3).

**Table 3:** Coexistence of resistance genes with integrons among carbapenem-resistant *P. mirabilis* isolates

Class / Gene	intI1 only *n* = 30 (52.6%)	intI2 only *n* = 5 (8.8%)	*intI1* + *intI2* *n* = 22 (38.6%)	*P* value
Class A (Serine β-lactamases)				
*bla*_PER_	10 (58.8%)	3 (17.6%)	4 (23.5%)	0.15
*bla*_VEB_	20 (60.6%)	3 (9.1%)	10 (30.3%)	0.3
*bla*_GES_	0	2 (100.0%)	0	0.000*
*bla*_KPC_	0	0	0	–
Class B (Metallo-β-lactamases, MBLs)				
*bla*_VIM_	20 (44.4%)	5 (11.1%)	20 (44.4%)	0.05
*bla*_NDM_	4 (28.6%)	2 (14.3%)	8 (57.1%)	0.1
*bla*_SIM_	0	3 (33.3%)	6 (66.7%)	0.001*
*bla*_GIM_	20 (76.9%)	0	6 (23.1%)	0.002*
*bla* ^IMP−1^	2 (33.3%)	0	4 (66.7%)	0.2
Class D (OXA-type carbapenemases)				
*bla*_OXA−23_	12 (60.0%)	2 (10.0%)	6 (30.0%)	0.6
*bla*_OXA−48_	6 (35.3%)	3 (17.6%)	8 (47.1%)	0.1
AmpC β-lactamase				
*bla*_FOX_	2 (50.0%)	0	2 (50.0%)	0.7

* (significant P value)

In this study, a high rate of *P. mirabilis* isolates carrying multiple carbapenemase-encoding genes was observed. As shown in Table 4, co-carriage of three or more carbapenemase genes was detected in 37 out of 57 carbapenem-resistant (CR) isolates (64.9%). All multi-gene isolates were classified as MDR, with a statistically significant association (*p* = 0.0001). Of these 37 isolates, 28 (75.7%) were recovered from inpatients and 9 (24.3%) from outpatients. Notably, all multi-gene isolates harbored integrons: *intI1* was present in 32 isolates (86.5%), while *intI2* was detected in 17 (45.9%). Additionally, the isolates from catheterized patients exhibited a higher frequency of carrying three or more carbapenemase genes.

The most frequently observed gene combination among carbapenem-resistant *P. mirabilis* isolates was the co-existence of *bla*_VIM1_ and *bla*_VEB_, detected in 29 isolates. Other notable combinations showing statistically significant correlations included *bla*_VEB_ and *bla*_GIM_, co-detected in 20 isolates (*r* = 0.353, *p* < 0.01), and *bla*_OXA−23_ with *bla*_VEB_ in 16 isolates (*r* = 0.329, *p* < 0.05).

**Table 4:** Co-carriage of carbapenemase-encoding genes among carbapenem-resistant *Proteus mirabilis* isolates

No. of coexisting CR genes	No. of isolates	MDR	Non MDR	Integron		Amp C	Outpatient	Inpatient	Catheterization	
				intI 1	intI 2				yes	No
1 gene	8	4	4	8	2	0	0	8	4	5
2 genes	12	12	0	12	8	0	2	10	8	4
3 genes	12	12	0	12	6	2	2	10	6	6
4 genes	12	12	0	10	4	0	4	8	4	8
5 genes	9	9	0	6	3	2	3	6	6	3
6 genes	0	0	0	0	0	0	0	0	0	0
7 genes	4	4	0	4	4	0	0	4	4	0
Multiple ≥ 3 genes	37	37	0	32	17	4	9	28	20	17

MDR (multidrug resistant)

## Discussion

*P. mirabilis* is a leading cause of UTIs. Although carbapenems are typically used for ESC-resistant infections, the rise of carbapenem-resistant *Proteus* species is becoming a significant public health threat [4]. In the present study, *P. mirabilis* occurred in 16.8% of urine samples, with a higher rate in inpatients (23.6%) than outpatients (8.5%). Comparable findings from recent Egyptian studies underscore the growing spread of *P. mirabilis* species in clinical settings, raising concerns about their increasing prevalence in hospitals over the past few years [14, 15]. Over half of clinical *P. mirabilis* isolates (57/101, 56.4%) from UTIs were resistant to imipenem, with nearly all CR isolates were MDR (93.0%). This alarming prevalence of carbapenem resistance in *P. mirabilis* underscores a significant clinical and epidemiological threat. Comparable rates of carbapenem resistance among *P. mirabilis* have been reported in Egypt, underscoring the escalating threat posed by MDR *Enterobacterales* [15–17]. High carbapenem resistance levels have been documented in other regions, including Saudi Arabia, Iraq, Pakistan, and China [18–21]. Furthermore, a recent systematic review and meta-analysis from Asia reported a significant rise in carbapenem resistance over the past decade [22]. This variation may reflect local carbapenem use, hospital-specific outbreaks of resistant strains, selective inclusion of isolates from hospitalized patients, or differences in infection control and laboratory practices.

The majority of CR isolates were obtained from inpatients (80.7%), emphasizing the role of hospitalization and healthcare exposure as as key risk factors for acquiring resistant strains. Additionally, urinary catheterization was prevalent among CR cases (56.1%), consistent with the established role of catheters in facilitating *P. mirabilis* colonization and persistence [23]. Interestingly, CR isolates were more prevalent among female patients (71.9%), including pregnant women, which is of clinical significance given the limited therapeutic options available during pregnancy. This could be attributed to their increased biological susceptibility to UTIs and the specific healthcare practices within gynecology and obstetrics departments, which contributed notably to the CR caseload. The highest frequency of CR isolates was found in urology (29.8%), gynecology (28.1%), and intensive care units (21.1%), all of which are high-risk settings where invasive procedures and frequent antibiotic use create selective pressure for resistance.

Antimicrobial susceptibility analysis revealed significantly higher resistance rates among CR isolates compared to carbapenem-sensitive ones, particularly against ceftazidime, aztreonam, and gentamicin. Notably, carbapenem resistance was significantly associated with resistance to third-generation cephalosporins such as ceftazidime and monobactams like aztreonam. This co-resistance pattern has been previously reported by Salama et al. (2025) in other regions of Egypt [24], as well as in countries like India [25]. These associations suggest cross-resistance between carbapenems and multiple antibiotic classes, likely driven by the co-location of resistance genes on mobile genetic elements, such as integrons and plasmids [26].

Nevertheless, aminoglycosides, particularly gentamicin and amikacin, retained partial activity against CR isolates, highlighting their potential role in combination therapy. This finding is consistent with previous studies, which have identified aminoglycosides as one of the few remaining treatment options against carbapenemase-producing *P. mirabilis* [15].

Molecular screening of CR *P. mirabilis* revealed a high prevalence of carbapenemase-encoding genes, with Class B MBLs being the most dominant. Bontron et al. highlighted *bla*_VIM−1_ as a key contributor to elevated carbapenem resistance in *P. mirabilis* [27]. The predominance of *bla*_*VIM−1*_ (78.9%) is consistent with a recent global meta-analysis, which reported that VIM-type enzymes are frequently detected in *Enterobacterales* and play a significant role in carbapenem resistance [22]. Additionally, Girlich et al. (2020) observed a rising prevalence of VIM-like MBLs and OXA-48 variants in *P. mirabilis* over time [3]. In Egypt, Shaaban et al. (2022) documented the presence of *bla*_VIM−1_ in 50% of carbapenemase-producing *P. mirabilis* clinical isolates [15]. The *bla*_*VIM−1*_ gene has also been identified previously among carbapenem-resistant *Enterobacteriaceae* and *Pseudomonas aeruginosa*, further supporting its widespread dissemination [28, 29]. Researchers identified *P. mirabilis* strains in which VIM-1 carbapenemase was located on the chromosome as part of a class 1 integron. This may explain the high prevalence of *bla*_VIM_ among the studied isolates [27]. Notably, *bla*_*VIM−1*_ is often carried on plasmids and associated with mobile genetic elements such as transposons, insertion sequences, and class 1 integrons, which collectively facilitate horizontal gene transfer and promote its rapid spread among bacterial populations.

Detection of bla_*NDM*_ in nearly one-quarter of isolates (24.6%) is particularly concerning, as this gene is widely disseminated in the region and associated with extensive resistance to β-lactams [16]. Moreover, another study reported bla_NDM−1_ in emerging extensively drug-resistant and multidrug-resistant *P. mirabilis* isolates [30]. A recent study from Tunisia reported an outbreak of *NDM*-producing *P. mirabilis* in Africa, underscoring a growing regional threat. The acquisition of *bla*_*NDM−1*_ in this intrinsically resistant species is particularly concerning, as it further limits treatment options by inactivating most β-lactams, including carbapenems, leaving few alternatives such as aztreonam [31]. A recent review by Bedenić and colleagues reported that the first carbapenemases identified in *Proteus* species belonged to class B, with *bla*_VIM−1_ and *bla*_NDM_ being the most frequently observed [32].

Among Class A carbapenemases, *bla*_VEB_ (57.9%) and *bla*_PER_ (29.8%) were the most frequently identified in *P. mirabilis*, while *bla*_GES_ was rare (3.5%) and *bla*_KPC_ was not detected. The relatively high prevalence of *bla*_VEB_ and *bla*_PER_ is significant, as these genes are often carried on mobile integrons, which can facilitate the co-selection of multidrug resistance. Studies have shown that *KPC*-type carbapenemases remain relatively uncommon in *P. mirabilis* compared to *Klebsiella pneumoniae* and *Escherichia coli* [33].

Class D carbapenemase genes *bla*_OXA−23_ and *bla*_OXA−48_ were identified in 35.1% and 29.8% of isolates, respectively. The *bla*_OXA−23_ gene was more commonly found in isolates exhibiting intermediate levels of resistance. Similarly, Potron et al. reported OXA-23-producing *P. mirabilis* strains in the French community that were still classified as carbapenem-susceptible, with inhibition zone diameters near clinical breakpoints [34]. Because of their low-level resistance, this clone is likely to go undetected in standard microbiological diagnostics. Interestingly, in this study, *bla*_OXA−48_ was significantly more prevalent among outpatient isolates. The detection of *bla*_OXA−48_ in *P. mirabilis* is rising and is considered an emerging public health concern due to its often-hidden resistance in the community [35]. This allows *P. mirabilis* to act as a silent reservoir for OXA-48-like genes, which can be acquired via conjugative plasmids and potentially transferred to other *Enterobacterales* species.

Only 29.6% of phenotypically AmpC-positive *P. mirabilis* isolates were confirmed by PCR, with *bla*_FOX_ being the only detected gene. Similarly, Shaaban et al. identified *bla*_FOX_ in 25% of isolates, along with other AmpC variants (*bla*_AmpC_, *bla*_ACC_, *bla*_ACT_), which were not examined in this study [15]. Also, this discrepancy highlights the complexity of cefoxitin resistance, which is not solely attributed to AmpC β-lactamase production. Other mechanisms, including ESBL or MBL production, porin mutations, and efflux pump activity, may also contribute [36]. Additionally, the presence of carbapenemase genes in 50% of *bla*_FOX_ -positive isolates suggests synergistic resistance mechanisms that could further complicate treatment.

In the present study, phenotypic ESBL production was detected in 38.6% of *P. mirabilis* isolates, with the *bla*_TEM_ gene being more prevalent than *bla*_SHV_ at the genotypic level. A previous study in Egypt reported 28.3% phenotypic ESBL-producing *P. mirabilis* isolates, with *bla*_TEM_ as the most common gene, followed by *bla*_SHV_ [14]. In contrast, another study conducted in Egypt observed a higher ESBL production rate of 51.7% among *P. mirabilis* isolates. Moreover, a recent meta-analysis estimated that approximately 46% of *P. mirabilis* strains are ESBL producers [15, 37]. These findings suggest an increasing trend in ESBL production over time, likely due to the widespread use of third-generation cephalosporins in hospital settings. In our study, the prevalence of ESBL genes among carbapenem-resistant isolates was relatively low, with *bla*_TEM_ and *bla*_SHV_ detected in 12.3% and 3.5% of carbapenem-resistant *P. mirabilis* strains, respectively. Consistently, Bedenić and colleagues highlighted the rapid evolution of β-lactam resistance mechanisms in *Proteus* species, progressing from extended-spectrum and plasmid-mediated AmpC β-lactamases to the emergence of carbapenemases [32].

The present study demonstrates a high prevalence of integrons among CR *P. mirabilis* isolates, emphasizing their pivotal role in horizontal gene transfer among our *P. mirabilis* isolates. Integrons act as genetic platforms that capture, integrate, and express gene cassettes, including those conferring antimicrobial resistance. Their widespread presence likely facilitates the transfer of carbapenemase and other resistance genes between bacteria, contributing to the dissemination of multidrug resistance in clinical settings. Class 1 integrons (*intI1*) were predominantly detected (91.2%), followed by class 2 integrons (*intI2*) at 47.4%, whereas class 3 integrons were absent. Importantly, all carbapenem-resistant isolates harbored at least one integron, indicating a strong association between integron presence and carbapenem resistance in *P. mirabilis* [38]. A statistically significant association was observed between *intI1* and inpatient isolates, as well as between *intI2* and both inpatient status and catheterization, underscoring their involvement in nosocomial transmission of resistance. These findings corroborate previous reports that highlight the global predominance of class 1 integrons as principal reservoirs of antimicrobial resistance genes [39]. Furthermore, they are consistent with data from Egypt demonstrating the critical role of integrons in facilitating the horizontal transfer of carbapenemase and other resistance determinants [40].

Integrons play a key role in multidrug resistance, with *intI1* and *intI2* detected in 86.5% and 45.9% of multi-gene carriers, respectively, highlighting their function as reservoirs for accumulating resistance genes. Notably, isolates from catheterized patients exhibited higher multi-gene carriage, likely due to selective pressure from prolonged antibiotic use and biofilm-mediated horizontal gene transfer in healthcare settings [41]. There were notable links between certain carbapenemase genes and types of integrons. Specifically, *bla*_GES_ was only found in isolates carrying class 2 integrons (*intI2*), suggesting that these integrons may help spread this gene. These results reinforce the role of integrons not only as genetic platforms for resistance but also in promoting the co-localization and maintenance of diverse β-lactamase genes within bacterial populations [42].

The findings of this study highlight an alarming trend of multi-gene carriage, with nearly 65% of carbapenem-resistant isolates carrying three or more carbapenemase genes, all exhibiting multidrug resistance. The co-existence of multiple carbapenemase genes is commonly observed in *Acinetobacter baumannii* and members of the *Enterobacteriaceae* family, especially *Klebsiella pneumoniae*, and has also been reported in *P. mirabilis* [25, 43]. This genetic redundancy enhances resistance by providing overlapping enzymatic activity against β-lactam antibiotics, thereby limiting therapeutic options. The significant association between multi-gene carriage and the multidrug-resistant phenotype underscores the clinical risks posed by these isolates, corroborating evidence that multi-gene carriage contributes to treatment failure and increased mortality in carbapenem-resistant *Enterobacteriaceae* [44].

Certain resistance gene combinations were notably prevalent. The co-occurrence of *bla*_*VIM−1*_ and *bla*_VEB_ in 29 isolates indicates synergistic spread of class B and class A β-lactamases. Significant correlations between *bla*_VEB_ and *bla*_GIM_, as well as *bla*_OXA−23_ and *bla*_VEB_, highlight frequent co-mobilization of diverse carbapenemase families, likely facilitated by integron capture and plasmid co-transfer [45].

Overall, these findings emphasize the dual challenge of integron-driven dissemination and multi-gene carriage in carbapenem-resistant *P. mirabilis*. The high prevalence of class 1 and 2 integrons underscores their critical role as genetic reservoirs promoting the persistence and spread of carbapenemases in both hospital and community settings. The lack of clonal typing, mainly due to financial constraints, limits interpretation to determine whether resistance spread occurred through horizontal gene transfer or through expansion of specific high-risk clones. This limitation should be addressed in future studies to better understand transmission dynamics.

## Conclusion

This study reveals a high prevalence of carbapenem resistance among *P. mirabilis* isolates from UTIs in Egypt, with most resistant strains exhibiting multidrug resistance. Class B metallo-β-lactamases (*bla*_VIM−1_, *bla*_NDM_) were predominant, alongside significant contributions from Class A (*bla*_VEB_, *bla*_PER_) and Class D (*bla*_OXA−23_, *bla*_OXA−48_) enzymes. Integrons, particularly class 1 and 2, were widespread and closely linked to carbapenemase gene carriage, underscoring their role in horizontal gene transfer. The frequent co-occurrence of multiple carbapenemase genes, especially in inpatient and catheterized patients, highlights the dual challenge of integron-driven dissemination and multi-gene accumulation in multidrug resistance. Uniquely, this study is among the first in Egypt to comprehensively investigate the coexistence of diverse carbapenemase genes alongside integron distribution, while also evaluating the contribution of catheterization to the spread of resistant *P. mirabilis* strains in hospital settings. These findings emphasize the urgent need for enhanced infection control, antimicrobial stewardship, and ongoing molecular surveillance to curb the spread of carbapenem-resistant *P. mirabilis* in healthcare and community settings.

## Supplementary Information

Below is the link to the electronic supplementary material.

Supplementary Material 1

## Data Availability

All data generated or analyzed during this study are included in this article.

## Abbreviations

CLSI: Clinical and Laboratory Standards Institute
CR: Carbapenem-resistant
EDTA: Ethylenediaminetetraacetic acid
ESBLs: Extended-spectrum beta-lactamases
ESC: Expanded-spectrum cephalosporins
GES: Guiana extended spectrum
GIM: German imipenemase MBL
ICU: Intensive care unit
IMP: Imipenemase
KPC: Klebsiella pneumoniae carbapenemase
MBL: Metallo-β-lactamase
MDR: Multiple drug-resistant
NDM: New Delhi metallo-β-lactamase
OXA: Oxacillinase
P. mirabilis: Proteus mirabilis
PER: Pseudomonas Extended Resistant
SIM: Seoul imipenemase MBL
UTI: Urinary tract infections
VEB: Vietnam Extended Spectrum β-lactamase
VIM: Verona integron-encoded metallo-β-lactamase
