# Supplementary material for: Dissemination of integrons and carbapenemase-encoding genes among multidrug resistant Proteus mirabilis isolated from urinary tract infections in Egypt
Source: BMC Infect Dis. 2026 Jan 27;26:179. doi: 10.1186/s12879-025-12447-4 (PMC12849061; doi:10.1186/s12879-025-12447-4)
Supplement: Supplementary file 1 — Supplementary Material 1 [file 12879_2025_12447_MOESM1_ESM.pdf]

Dissemination of Integrons and Carbapenemase-encoding genes among multidrug resistant *Proteus mirabilis* isolated from Urinary tract infections in Egypt

Table S1: PCR primers used in this study and their expected amplicon sizes:

Primer	Primer sequence (5' to 3')	Amplicon size (bp)	Annealing Temperature	Reference
***bla*TEM**	F-ATGAGTATTCAACATTTCCG<br>R-CCAATGCTTAATCAGTGAGG	832	51°C	1
***bla*SHV**	F-CGCCGGGTTATTCTTATTTGTCGC<br>R-TCTTTCCGATGCCGCCGCCAGTCA	1016	55°C	
***bla*PER**	F: AATTTGGGCTTAGGGCAGAA<br>R: ATGAATGTCATTATAAAAGC	933	50°C	2
***bla*VEB**	F: CGACTTCCATTTCCCGATGC<br>R: GGACTCTGCAACAAATACGC	642	55°C	3
***bla*IMP1**	F: CTACCGCAGCAGAGTCTTTG<br>R: AACCAGTTTTGCCTTACCAT	587	55°C	4
***bla*VIM1**	F: AGTGGTGAGTATCCGACAG<br>R: ATGAAAGTGCGTGGAGAC	261	55°C	5
***bla*SIM**	F: TACAAGGGATTCGGCATCG<br>R: TAATGGCCTGTTCCCATGTG	570	52°C	6
***bla*GIM**	F: TCGACACACCTTGGTCTGAA<br>R: AACTTCCAACTTTGCCATGC	477	52°C	7
***bla*NDM**	F: GGTTTGGCGATCTGGTTTTC<br>R: CGGAATGGCTCATCACGATC	621	52°C	
***bla*OXA-48**	F: GCGTGGTTAAGGATGAACAC<br>R: CATCAAGTTCAACCCAACCG	438	55°C	
***bla*KPC**	F: CGTCTAGTTCTGCTGTCTTG<br>R: CTTGTCATCCTTGTTAGGCG	798	55°C	
***bla*GES**	F: ATGCGCTTCATTCACGCAC<br>R: CTATTTGTCCGTGCTCAGG	864	55°C	8
***bla*OXA-23**	F: GATCGGATTGGAGAACCAGA<br>R: ATTTCTGACCGCATTTCCAT	501	50°C	9
***bla*MOX**	F-5' GCTGCTCAAGGAGCACAGGAT-3'<br>R-5' CACATTGACATAGGTGTGGTGC-3'	520	64°C	10
**blaCIT**	F-5' TGGCCAGAACTGACAGGCAAA-3'<br>R-5' TTTCTCCTGAACGTGGCTGGC-3'	462		
***bla*DHA**	F-5' AACTTTCACAGGTGTGCTGGGT-3'<br>R-5' CCGTACGCATACTGGCTTTGC-3'	405		
***bla*FOX**	F-5' AACATGGGGTATCAGGGAGATG-3'<br>R-5' CAAAGCGCGTAACCGGATTGG-3'	190		
intI1	F-5′ CAGTGGACATAAGCCTGTTC-3′<br>R-5′ CCCGAGGCATAGACTGTA-3′	160	55°C	11
intI2	F-5′ GTAGCAAACGAGTGACGAAATG-3′<br>R-5′ CACGGATATGCGACAAAAAGGT-3′	789	55°C	
intI3	F-5′ GCCTCCGGCAGCGACTTTCAG-3′<br>R-5′ ACGGATCTGCCAAACCTGACT-3′	979	55°C	

Abbreviations: bp (base pair)

References

1. Tonkić M, Mohar B, Šiško-Kraljević K, et al. High prevalence and molecular characterization of extended-spectrum β-lactamase-producing Proteus mirabilis strains in southern Croatia. J Med Microbiol. 2010;59(Pt 10):1185-1190. doi:10.1099/jmm.0.016964-0
2. Claeys, G., G. Verschraegen, T. De Baere, and M. Vaneechoutte. PER-1 β-lactamase producing Pseudomonas aeruginosa in an intensive care unit. J. Antimicrob. Chemother.2000, 45:924-925. https://doi.org/10.1093/jac/45.6.924
3. Poirel, L., Rotimi, V. O., Mokaddas, E. M., Karim, A., & Nordmann, P. VEB-1-like extended-spectrum beta-lactamases in Pseudomonas aeruginosa, Kuwait. Emerging infectious diseases. 2001a, 7(3), 468–470. https://doi.org/10.3201/eid0703.010322
4. Senda K, Arakawa Y, Ichiyama S, et al. PCR detection of metallo-beta-lactamase gene (blaIMP) in gram-negative rods resistant to broad-spectrum beta-lactams. J Clin Microbiol. 1996;34(12):2909-2913. doi:10.1128/jcm.34.12.2909-2913.1996
5. Tsakris A, Pournaras S, Woodford N, et al. Outbreak of infections caused by Pseudomonas aeruginosa producing VIM-1 carbapenemase in Greece. J Clin Microbiol. 2000;38(3):1290–1292. doi: 10.1128/JCM.38.3.1290-1292.2000
6. Lee K, Yum JH, Yong D, et al. Novel acquired metallo-beta-lactamase gene, bla(SIM-1), in a class 1 integron from Acinetobacter baumannii clinical isolates from Korea. Antimicrob Agents *Chemother. 2005;49(11):4485-4491. doi:10.1128/AAC.49.11.4485-4491*.2005
7. Poirel L., Walsh T.R., Cuvillier V., Nordmann P. Multiplex PCR for detection of acquired carbapenemase genes. Diagn. Microbiol. Infect. Dis. 2011; 70:119–123. doi: 10.1016/j.diagmicrobio.2010.12.002.
8. Poirel, L., Weldhagen, G. F., Naas, T., De Champs, C., Dove, M. G., & Nordmann, P. GES-2, a class A beta-lactamase from Pseudomonas aeruginosa with increased hydrolysis of imipenem. Antimicrobial agents and chemotherapy. 2001b; 45(9), 2598–2603. https://doi.org/10.1128/AAC.45.9.2598-2603.2001
9. Woodford, N.; Ellington, M.J.; Coelho, J.M.; Turton, J.F.; Ward, M.E.; Brown, S.; Amyes, S.G.; Livermore, D.M. Multiplex PCR for genes encoding prevalent OXA carbapenemases in Acinetobacter spp. Int. J. Antimicrob. Agents. 2006, 27, 351–353. https://doi.org/10.1016/j.ijantimicag.2006.01.004
10. Pérez-Pérez FJ, Hanson ND. Detection of plasmid-mediated AmpC beta-lactamase genes in clinical isolates by using multiplex PCR. J Clin Microbiol. 2002; 40(6):2153-2162. doi:10.1128/JCM.40.6.2153-2162.2002
11. Dillon B, Thomas L, Mohmand G, Zelynski A, Iredell J. Multiplex PCR for screening of integrons in bacterial lysates. J Microbiol Methods. 2005; 62(2):221-232. doi:10.1016/j.mimet.2005.02.007
